# *‘‘That's just part of having a transplant, that's the price you pay”* Patient-reported facilitators and barriers to immunosuppressant medication adherence among transplant recipients: a qualitative study

**DOI:** 10.1080/21642850.2026.2641868

**Published:** 2026-03-12

**Authors:** Cheyenne Downey, Aisling Walsh, Frank Doyle, Peter Conlon, Conall O'Seaghdha, P. Aiden McCormick, Audrey Dillon, Peter Riddell, Lisa Mellon

**Affiliations:** a Department of Health Psychology, School of Population Health, RCSI University of Medicine and Health Sciences, Dublin, Ireland; b Department of Public Health and Epidemiology, School of Population Health, RCSI University of Medicine and Health Sciences, Dublin, Ireland; c Department of Nephrology, Beaumont Hospital, Dublin, Ireland; d Department of Hepatology, St. Vincent's University Hospital, Dublin, Ireland; e School of Medicine, University College Dublin, Dublin, Ireland; f Department of Respiratory Medicine, Mater Misericordiae University Hospital, Dublin, Ireland

**Keywords:** Medication, adherence, transplant recipients, immunosuppressive therapy, immunosuppressants

## Abstract

**Introduction:**

It is estimated that 1 in 5 organ transplant patients fail to properly adhere to their immunosuppressant medication. Interventions for good adherence lack efficacy, and challenges to developing interventions tailored to individual patient needs remain. This study explored patient-reported facilitators and barriers to immunosuppressant medication adherence, and patient-recommended strategies to support good adherence. The findings were mapped to the ABC Taxonomy for medication adherence to provide a framework to guide future intervention development.

**Methods:**

Semi-structured interviews were held with heart, lung, liver and kidney transplant recipients (*n *= 16) who were taking prescribed immunosuppressant medication for a minimum of 6 months post-transplant. Inductive thematic analysis was conducted to identify key themes.

**Results:**

Themes identified were: (1) the psychological processes impacting medication adherence, including lack of post-transplant psychological support, peer support from fellow transplant recipients and reflecting on health improvements following transplantation; (2) the practicalities of adhering to medication, including in-hospital medication training, routine implementation, managing domestic and social responsibilities and side effect experiences; and (3) patient-recommended strategies to facilitate medication adherence among newly transplanted patients, including mobile apps and alarms, visual reminders and pillboxes. The Persistence phase of the ABC Taxonomy represented intentional non-adherence related barriers to longer-term medication adherence, with emphasis on the importance of accessing psychological support to promote lifelong adherence.

**Conclusion:**

Themes mapped to the ABC Taxonomy for medication adherence highlighted that future interventions should focus on intentionally non-adherent patients and address the complex physical, cognitive and emotional themes influencing longer-term persistence to the immunosuppressant medication regimen.

## 1. Introduction

Solid organ transplant recipients are required to take prescribed immunosuppressive medication for the duration of graft survival. Maintenance medication regimens are broadly consistent across types of organ transplant in the first 1–2 years after transplantation and usually include triple drug therapy in the form of calcineurin inhibitor(s), corticosteroids and an antimetabolite (Hussain et al., 2021). Some regimens may decrease to mono- or dual agents in liver and kidney transplants in subsequent years post-transplant (Low et al., 2015). Adherence is defined as a dynamic health behaviour whereby patients take medications as prescribed by the healthcare provider (Low et al., 2015; Nieuwlaat et al., 2014). The Ascertaining Barriers to Compliance (ABC) taxonomy for measuring medication adherence describes adherence behaviour as comprising three distinct but interrelated stages—Initiation, Implementation and Persistence (De Geest et al., 2018; Michie et al., 2013; Vrijens et al., 2012, 2016). Initiation involves starting a medication regimen. Implementation requires taking medications as prescribed, including the correct dosage and timing, and avoiding drug holidays. Persistence is a continuation of correct medication taking for the prescribed duration; in the case of organ transplantation, persistence represents an indefinite duration.

Meta-analyses of all solid organ transplant types report a 22.6% non-adherence rate to immunosuppressant medication (Mellon et al., 2017). Rates of non-adherence vary by type of transplant, with the highest rates observed for kidney transplants (36%) and the lowest for liver transplants (6%) (Dew et al., 2007). The reasons for non-adherence are multifaceted and individualised (Tang et al., 2021) and can be ‘intentional’ or ‘non-intentional’. Intentional non-adherence involves deliberately deciding to alter or abstain from the prescribed regimen (Griva et al., 2018), such as feeling physically well, the burden of side effects, procrastination, poor patient‒doctor relationships or financial restrictions (Mukhtar et al., 2014). Non-intentional non-adherence can arise from forgetting medication and poor understanding of instructions or administering incorrect doses (DiMatteo et al., 2012). Non-intentional non-adherence has also been attributed to poor understanding of the physiological importance of immunosuppressants (Bendersky et al., 2023; Stuber, 1993). For accurate implementation of the medication regimen, correct ‘taking’ and ‘timing’ of the drug is essential (Springfield-Trice et al., 2025). The timing of drug administration is of particular importance post-transplant for correct drug absorption (Araya & Tasnif, 2023), with non-adherence to the timing of the drug regimen associated with acute rejection episodes (Corr et al., 2025).

Meta-analysis of interventions to improve adherence to immunosuppressants showed that there was some limited evidence that interventions can be effective in improving taking the correct dosage of immunosuppressants, however overall, the evidence was of poor methodological quality; thus, the quality of available evidence is currently insufficient to guide practitioners working with post-transplant patients (Mellon et al., 2022; Springfield-Trice et al., 2025).

The variability in the type of adherence assessment can affect the quality of assessment across different phases of adherence behaviour (Nieuwlaat et al., 2014). Objective, direct measures of adherence to medication regimens post-transplant, such as direct observation, pharmacy refill data, or electronic measurement of pillbox use (Denhaerynck et al., 2005), allow accurate measurement of taking and timing adherence, in addition to persistence. Indirect proxy measures of medication adherence, including self-report assessments, yield superior rates of medication adherence; however, they are open to considerable reporting biases (Moran et al., 2017). Notably, generic self-reported medication adherence tools do not accurately capture all phases of adherence; however, transplant-specific measures, such as the BAASIS tool, capture taking and timing adherence, in addition to drug holidays and persistence (Denhaerynck et al., 2023). Objective, indirect surrogate measures of immunosuppressant medication adherence, such as assay of medication or fluctuation in medication trough levels (O'Regan et al., 2016), and/or clinical outcomes, such as rejection or organ failure, can indicate a clinical outcome of non-adherence, but do not yield information about adherence behaviour itself.

Non-adherence is associated with a seven-fold increase in the odds of organ failure (Butler et al., 2004; Pinsky et al., 2009), so designing interventions to improve adherence should accurately assess the behaviour that is driving non-adherence. The most effective interventions to improve medication adherence and reduce the risk of non-adherence post-transplant should be designed by firstly asking the patient what works for them rather than starting with clinical judgement of what works best for patients (Dew & DeVito Dabbs, 2016). Previous qualitative research reported that patients were concerned about the side effect burden on quality of life but were motivated to take their immunosuppressants to prevent rejection, to preserve their freedom and to prevent further health complications (Tang et al., 2021). Our study investigated immunosuppressant medication experiences amongst all solid organ transplant recipients to identify and address collective and group-specific challenges with immunosuppressant medication adherence. Exploration of medication adherence experiences among various organ transplant recipients (heart, lung, liver and kidney) has helped identify factors that may be unique to different organ groups in influencing how they adhere to immunosuppressive therapy. Understanding determinants of medication adherence among each organ group may be especially beneficial in informing future developments that aspire to provide tailored post-transplant care or resources to promote good medication adherence. Further, our aim was to identify patient-preferred and applied strategies for good adherence and recommendations for newly transplanted patients and map the identified challenges and strategies to the ABC taxonomy for medication adherence to help guide the development of a future intervention to improve overall adherence to immunosuppressant medication.

## 2. Methods

### 2.1. Study design

This study employed a descriptive qualitative research design (Kim et al., 2017) to investigate patient-preferred strategies for medication adherence post-organ transplant and to examine patient-reported facilitators and barriers to medication adherence post-transplant. Findings are reported in accordance with the COREQ checklist for qualitative research (Tong et al., 2007). Ethics approval was granted by the Research Ethics Committees of RCSI University of Medicine and Health Sciences (Reference: REC 202210027), St. Vincent's University Hospital (Reference: RS23-035) and the Mater Misericordiae University Hospital (Reference: 1/378/2357) in Ireland.

### 2.2. Participants and setting

The inclusion criteria were adult solid organ transplant recipients (heart, lung, liver, kidney), who were aged 18 years or older and taking prescribed immunosuppressants for a minimum of 6 months. The reason for including all solid organ transplant recipients was to identify any similarities and differences in how they experienced immunosuppressive therapy and medication adherence. The transplant physicians in each hospital supported participant recruitment. Using purposive sampling, potential candidates attending out-patient clinics were informed about the study and given a participant information leaflet containing contact details of the study researcher, CD. Recruitment was also facilitated by advertisements on social media channels of four Irish charities supporting transplant patients: the Irish Kidney Association, the Irish Liver Foundation, the Irish Heart and Lung Transplant Association and the Irish Lung Fibrosis Association. Snowball sampling was applied simultaneously, whereby existing participants informed potential participants about the research and invited them to contact CD. Semi-structured interviews were conducted by CD between December 2023 and May 2024. Thirteen semi-structured interviews were conducted via MS Teams and three by phone.

### 2.3. Data collection

An interview schedule was developed to assess the study objectives (Supplementary Material 1), and pilot tested with patient support volunteers of the Irish Kidney Association. Open-ended questions pertaining to challenges and barriers to adhering to immunosuppressant medication, the experience of medication and its side effects, and strategies employed to adhere to the immunosuppressive regimen were included in the interview schedule. Questions were used as prompts for discussion and to ensure consistency across interviews whilst allowing flexibility for participants to express personal experiences.

Interviews lasted up to 40 min. All the discussions were audio-recorded and the interviews were transcribed verbatim. Online transcription was conducted using the Microsoft OneDrive transcription function, and phone transcription was conducted manually. Field notes were not documented. The participants were offered the opportunity to review their transcripts and any potentially identifying information was removed to preserve participants' confidentiality; no participant requested to view their transcript. All participants received a €50 multi-use voucher for their participation.

### 2.4. Data analysis

Reflexive inductive thematic analysis was carried out via NVivo 12. A six-step thematic framework (Braun and Clarke, 2006; Braun and Clarke, 2021a, 2021b) was used to analyse the findings: (1) CD, an experienced qualitative researcher, and LM, a psychologist with qualitative data analysis training, reviewed the transcripts for familiarisation. (2) CD and LM coded the data separately before meeting to discuss similarities and differences across different transplant types. Following discussion, the data underwent another round of coding by CD and LM. A meeting was then convened with a third study co-investigator AW, a qualitative methods expert, who reviewed preliminary codes and helped finalise the selected codes. (3) From this, the research team developed potential themes and subthemes (4). Next, potential themes and subthemes were modified. (5) Themes and subthemes were refined to correspond with the code descriptions. (6) Each theme was finalised and depicted using illustrative quotes from the data (Naeem et al., 2023). Reflexive practice was central to conducting the interviews and data analysis, as the data analysts (CD and LM) had family experiences with organ failure. Open dialogue and consideration of personal assumptions was therefore engaged throughout, with peer debriefing—including reflections on the patient-researcher dynamic—conducted throughout.

## 3. Results

Sixteen participants participated in semi-structured interviews. The sample comprised of 8 females and 8 males, all of whom were white Irish—7 liver (4 females, 3 males), 5 kidney (3 females, 2 males), 2 heart (2 males) and 2 lung transplant (1 female, 1 male) recipients, between the ages of 18–65+ years; however, no patients under the age of 25 participated in the study.

There is a five-year average of 238 organ transplants in Ireland (Organ Donation Transplant Ireland, 2020; Organ Donation Transplant Ireland, 2021; Organ Donation Transplant Ireland, 2022; Organ Donation Transplant Ireland, 2023), which represents a small cohort of patients attending outpatient post-transplant clinics nationally. Owing to concerns about the potential identifiability of participants by the institutional Research Ethics Committees of the included sites, minimal demographic data was collected and the data was recorded in bands rather than exact numbers. The majority of the participants were transplanted for 3–5 years (44%) and were between the ages of 35–44 years old (38%) and 45–54 years old (25%). All other age groups (25–34, 55–64 and 65+) each represented approximately 13% of the sample.

Three major themes were identified that reflected participants' experience of taking immunosuppressants across all organ types: (1) psychological processes that affect medication adherence; (2) the practicalities of adhering to medication; and (3) patient-recommended strategies to facilitate medication adherence.

### 3.1. Psychological processes – facilitators of medication adherence

#### 3.1.1. Patient reflection on pre-transplant health

Participants reported distinguishing the pros and cons of immunosuppressants as a self-help method for adherence; some reflected on their pre-transplant health as a reminder that their immunosuppressants are elongating their life. The participants also reported living with fewer restrictions despite adhering to immunosuppressive therapy, with kidney transplant recipients especially thankful for no longer requiring dialysis treatment, and other transplant recipients mindful of extended hospital admissions prior to transplantation:

“*The biggest thing that drives me to take my tablets every day is the fear of rejection. Like you know, I don't want to end up back in the [hospital], as sick as what I was before the heart transplant”* (Participant 16—Heart Transplant)

Fulfilling a duty of care to their organ donor was often highlighted as a rationale for continuing to take their immunosuppressants, even when experiencing debilitating side effects such as decreased kidney function among non-kidney transplant recipients:

“*I feel very protective of these lungs. That they were once somebody else’s and I feel like a custodian and I’m minding them on behalf of someone else and someone else’s family even more so.”* (Participant 17 - Lung Transplant)

#### 3.1.2. Having access to social support

Many participants emphasised that when struggling with adherence to a medication regimen, a conscious focus on what patients gain from receiving a transplant is essential and occurs when there is support from transplant peers or transplant patient support services to increase motivation for continued adherence:

“*If somebody says: ‘look, I've been there, I know exactly what's going on with you,’ it pulls a heck of a lot more weight than some person dressed in white.”* (Participant 3—Kidney Transplant)

Heart and liver patients reported disparities in access to support among them and kidney transplant recipients. Several participants stated that they have initiated informal contact with other patients, as there are no formal support services equivalent to the Irish Kidney Association for heart, lung and liver transplant patients in Ireland. Some detailed joining social media groups and arranging social events with fellow transplant recipients, which helped clarify the ‘do's and don'ts’ of immunosuppressive therapy and facilitate good adherence.

Participants underpinned that facilitating access to professional support associations, such as organised social events can help patients regulate negative emotions associated with the side effects of immunosuppressants:

*“You can feel like a freak at the start and angry and resentful. But, when you actually see there are people 18 years or 22 years post-transplant, you're like: ‘OK, this is just part of it. Just take it. Get to where they are’, you know.”* (Participant 12—Liver Transplant)

### 3.2. Psychological processes – barriers to medication adherence

#### 3.2.1. Poor access to psychological support services

The psychological toll of organ transplantation lacks clinical attention, with participants reporting that counselling/therapeutic interventions are needed by most patients to support the psychological processing of transplantation and its required lifestyle changes, including taking medications with significant side-effect profiles. The participants felt that the transplant team should prioritise directing patients to the appropriate mental health services to help patients come to terms with their post-transplant state. They further expressed that a lifelong reliance on immunosuppressive therapy can result in emotional distress and resentment, hindering adherence if the need for immunosuppressive therapy and other related challenges remain emotionally unprocessed. Participants were of the opinion that the transplant team should play a role in promoting adherence during all stages of the post-transplant journey, by facilitating access to appropriate mental health supports, including peer support services:

*“I went to the coordinators in [hospital] and I begged, I begged! and I said: ‘please, I need to talk to someone who's been through this, because I couldn't find any story that was like mine online and I was told no, there's no support group.”* (Participant 12—Liver Transplant)

Furthermore, there were discrepancies in the level of psychological care accessed by each organ group. For example, heart, lung and liver transplant recipients reported that they do not have access to free counselling unlike kidney transplant recipients in Ireland (provided by the Irish Kidney Association), with one lung transplant recipient stating: ‘*you get all the physical stuff, nothing for the head.’* Kidney transplant recipients who reported attending counselling via a patient support organisation, emphasised the importance of accessing professional psychological care post-transplant. Some participants mentioned that it was through emotional regulation that their attitudes towards immunosuppressant use and medication adherence improved.

### 3.3. Practical facilitators of medication adherence

#### 3.3.1. Intensive inpatient hospital training

All participants noted that the intensive training provided by transplant nurses and pharmacists post-transplant was vital for starting life-long medication adherence. Training consisted of written materials, practical medication training, verbal one-to-one consultations with an on-site pharmacist and twice-daily monitoring of medication self-administration on the ward. Opportunities to self-direct their care fostered a better understanding of the importance of taking immunosuppressants correctly to prevent further health complications and unplanned hospital admissions:

*“It's great because then they come in and say: ‘right, you're going to take your tablets today’, but they're watching. You say to them: ‘I need 2 mg of prograf, I need this, I need that’ and then you're trying not to forget anything. And then they say, right: ‘OK, brilliant, you've done it.’ And then it gets to stage where you ring the bell when you're due your medication.” So, if it's four times a day or whatever six times a day, you're ringing to say I need my medication. Now, if you don't ring, they come in of course and they say: ‘you didn’t ring!”* (Participant 8—Liver Transplant)

Some participants also reported that, in the initial post-transplant stages, their transplant team provided them with a journal to document their medication schedule daily, as a way of helping them take their medication persistently (and correctly). Once familiarised and accepting of their medication routine, participants said they stopped using the journal but acknowledged it as a good starting point post-transplant.

### 3.4. Practical barriers to medication adherence

#### 3.4.1. Disruptions to daily medication routine

Participants reported that following the immunosuppressant regimen correctly requires implementing a daily routine and planning events around their medication schedule; it was emphasised that distractions from their normal routine can disrupt their medication schedule. For example, when patients attend blood test appointments, they must not take their morning dose, which indirectly causes them to also miss their evening dose:

*“If I'm going to an appointment in [hospital], you think I would remember, I either take it and I know I'm having blood tests and shouldn't take it or I don't take it and then I don't remember until I'm almost home again on the train and it's like 7 hours late or whatever.”* (Participant 8—Liver Transplant)

Whilst participants reported occasionally missing a scheduled dose, no participant reported experiencing significant health complications that resulted in acute toxicity or acute rejection episodes due to non-adherence. Concerningly, some patients reported becoming gradually more lenient with the timing of their drug-taking, with long-term transplant recipients reporting being less ‘*regimental’* over time and said taking their tablets earlier or later than usual is ‘*not a problem’* once they are consumed sooner than later, which goes against clinical guidelines:

“*You wouldn't be doing it* [missing immunosuppressants] *every day, because obviously you're totally skewing things up there, but it's not a big deal if it's a day or once every now and* *then.*” (Participant 1—Kidney Transplant)

#### 3.4.2. Fitting the medication regimen into normal life

Participants outlined that family and social life responsibilities can be a barrier to how and when they take their medication . For example, participants with young families described difficulties aligning family mealtimes and domestic responsibilities with the timing of their medication.

Dining-out for social occasions was also a perceived barrier, as participants were often self-conscious of taking medication in the company of others or causing meal-time disruptions at celebratory events; consequently, some liver and kidney transplant participants reported abstaining from such events, whereas some heart transplant participants affirmed that it did not bother them to take their medication in front of others.

Moreover, participants in employment (compared to those not working) reported that it was difficult to not only establish a medication routine but also to remember to take their immunosuppressants, particularly when doing shift work (i.e. working outside of traditional business hours). Despite this barrier, they reported feeling motivated to return to work post-transplant and recognised adherence as a determinant of their ability to continue working:

“*I was very motivated to get back to work and back to life and you're not going to, if you don't take your immunosuppressants.”* (Participant 8—Liver Transplant)

#### 3.4.3. Side effect burden

All participants experienced significant side-effects from immunosuppressive therapy. Common side effects reported were shaking hands, upset stomach, brain fog, hair loss, bloating and fluid retention, which were often challenging to manage. There were minor differences in the side effects experienced across the different transplant types; however, reports of brain fog and shaking hands were most prevalent among liver transplant recipients. Kidney transplant recipients typically experienced an upset stomach, hair loss and facial bloating *(‘moon face’).* Fluid retention (including around the ankles), shaking hands, memory loss and an upset stomach commonly affect heart and lung transplant recipients.

Furthermore, the damaging side effect of decreased kidney function, often necessitating dialysis treatment or a kidney transplant was particularly distressing for non-kidney transplant recipients:

“*I now have stage 3 kidney disease as a result of those. So, it can be a hard pill to swallow, excuse the pun, because I had a perfect kidney pre-op.”* (Participant 17 - Lung Transplant)

However, it was stressed that failure to comply with medical advice will diminish initial efforts made for transplant eligibility, and some participants perceived the side-effect burden as the ultimate sacrifice for optimal transplant function.

### 3.5. Patient-recommended strategies to facilitate medication adherence

#### 3.5.1. Setting alarm reminders

Setting mobile phone alarms was the most common strategy used and recommended by participants to help them take their immunosuppressants as prescribed.

Participants explained that during their in-hospital medication training, the on-site pharmacist encouraged them to select a morning time slot and set an alarm for their immunosuppressants that would facilitate a reasonable time for taking their evening dose.

#### 3.5.2. Mobile apps for transplant recipients

Most kidney transplant recipients are referred to a transplant-specific app developed by their transplant team to help monitor and update their dose of immunosuppressants. This facilitated the familiarisation of medication by recording tablets and dosage instructions and provided timely, personalised nursing support with dosage updates and medication queries:

“*You can pick the colour and the shape and the size of your tablet and name it* [on the app], *which I just think for older or even colour blind, whatever the case may be, when you're on a new dosage of something, it really, really helps”* (Participant 1—Kidney Transplant)

#### 3.5.3. Visual exposure

Liver and lung transplant recipients specifically noted that they do not have a post-transplant medication reminder app and instead rely on their phone alarms and visual exposure to their daily immunosuppressants as a psychological trigger to take them. Placing their tablets on their bedside locker or beside kitchen appliances are commonly used strategies to facilitate adherence.

#### 3.5.4. Pill boxes

A complex medication regimen with a high pill burden can be difficult to follow, particularly with constant-dose alterations; therefore, patients emphasised that a pill box minimises the risk of error:

*“It can be confusing, you go: ‘Oh, God, did I take them or not?’ No problem, just check the pill box, if they're there, you didn't take them.”* (Participant 3 - Kidney Transplant)

#### 3.5.5. Ask questions, seek support and avoid self-labelling

Participants who were at least 3 years post-transplant recommended for new transplant recipients who may struggle with long-term medication adherence to ask questions and inform their transplant team of any issues with side effects to support their post-transplant wellbeing. The participants also advised new patients to associate their medication schedule with an existing everyday activity (e.g. dog walking) to facilitate good adherence. Seeking support from fellow transplant recipients and establishing a sense of self outside of their condition was also recommended to help normalise immunosuppressive therapy:

“*I think it helps to have an identity other than the illness. You know, it's really lovely that I can go to a room or speak at a conference and nobody knows anything about that stuff* [immunosuppressants].*”* (Participant 12—Liver Transplant)

### 3.6. Mapping of themes to the ABC taxonomy for medication adherence

**Figure 1.**
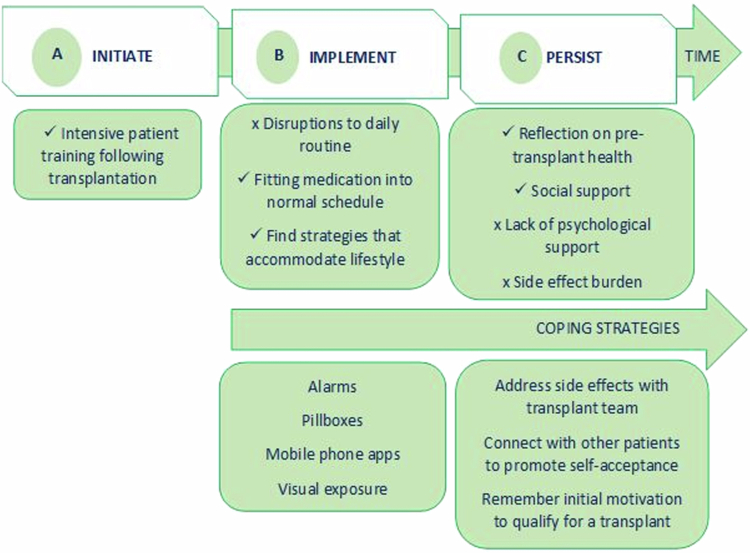
Mapping of themes to the ABC taxonomy for medication adherence.

Figure 1 depicts the facilitators and barriers to good adherence from the qualitative themes, as mapped onto the phases of medication taking from the ABC Taxonomy; Initiation, Implementation, and Persistence. The findings show that the implementation phase reflects ‘non-intentional’ non-adherence, as challenges relate to day-to-day medication taking, such as forgetting and schedule disruptions. The persistence phase, representing longer-term medication adherence over the life course of the transplant, is influenced by ‘intentional’ non-adherence, relating to more complex physical, cognitive, and emotional challenges that affect coping post-transplant, such as side effect burdens and a lack of psychological and social support. The participants highlighted that temptation for non-adherence due to physical and emotional burdens can be mitigated by having consistent access to transplant-specific resources, including peer support services and counselling, as the availability of such resources encourages persistence in adhering to their medication regimen despite personal challenges.

## 4. Discussion

Our results demonstrate a complex picture of adherence behaviour. Personal processes, including the psychological trauma associated with organ failure, a lack of social support, competing family responsibilities and side-effect burden affected consistent, intentional persistence over time to adhering to immunosuppressant medication. Patients provided guidance for newly transplanted patients, emphasising the importance of not allowing the transplant to dominate their self-identity. Mapping of themes to the ABC taxonomy for medication adherence highlighted the Persistence phase as an area of greatest concern, representing the more complex barriers related to intentional non-adherence that affect good adherence for the duration of the transplant (Vrijens et al., 2012).

Facilitators of good adherence during the Initiation and Implementation phase were personal, environmental and systems-based factors; incorporating professional support and informal peer supports, self-reflections on the benefits of transplantation; and in-hospital medication training post-transplant and supportive medication management devices and technology. Whilst ‘taking’ adherence was reported as stable over time, the ‘timing’ of drug administration was relaxed by some patients, particularly those who felt clinically well. For longer-term Persistence, mental health counselling, incorporating professional support and informal peer supports, facilitated longer-term adherence, emphasising the importance of the psychological support needed for transplant patients for the lifetime of the transplant, not just in the acute phase.

Whilst the number of transplants has increased two-fold in the past 30 years, there is a simultaneous six-fold increase in the numbers wait-listed for a transplant (Kupiec-Weglinski, 2022), indicating a worldwide shortage of organ donors. This underlines the critical importance of adherence to the immunosuppressant regimen to prevent organ rejection and the need for subsequent re-transplantation. Whilst tried and tested methods of medication pillboxes and alarm reminders were suggested by some patients in this study as useful strategies for correct adherence, mapping of facilitators and barriers to the ABC taxonomy for medication adherence identified that long-term medication adherence is affected by physical and psychosocial challenges. Better psychological support post-transplant to manage emotional struggles with lifelong medication and its significant side effects is desired by patients from their multidisciplinary team, in addition to opportunities for informal peer support from fellow transplant recipients. Access to professional patient support associations was also identified as a key strategy in coping with the medication regimen and its side effects, as participants felt that being able to avail of counselling and peer support helps them regulate overwhelming emotions post-transplant and, overall, reduces feelings of isolation by facilitating a connection with someone who knows what they are going through. While there are reportedly good systems in place for kidney and heart transplant participants. However, some inequity in access was highlighted by other organ groups, with lung and liver transplant participants in particular, reporting a lack of formal support in place for these kinds of interactions to occur. These qualitative findings concur with previous work on quantitative non-adherence in kidney transplant recipients.

These findings have clinical relevance; incorporation of routine mental health screening into post-transplant outpatient care represents an opportunity to identify psychological difficulties that may contribute to intentional non-adherence. Appropriate monitoring of physical and mental health during clinical appointments cultivates trust between patients and their transplant team, as patients are provided with an individualised assessment of their physical and mental health, tailored specifically to their wellbeing needs. Such appointments will also give the transplant team an opportunity to disclose appropriate and accurate information and/or refer patients to supports (e.g. peer support groups, counselling) that are in their best interest, in order to promote medication adherence and good quality of life. Early identification through structured screening enables timely intervention and support. Furthermore, referring patients to peer support groups or relevant charities may provide opportunities for enhancing coping tolerance and self-acceptance of their need for immunosuppressive therapy through shared lived experiences with other transplant patients.

Previous systematic reviews of qualitative studies addressing immunosuppressant medication adherence identify a similar range of barriers to good adherence (Jamieson et al., 2016; Tong et al., 2011), however barriers to good adherence have been reported to vary across transplant types (Colmenero et al., 2024; Denhaerynck et al., 2018; Hu et al., 2017; Hussain et al., 2021; Jamieson et al., 2016; Nevins et al., 2017; Nieuwlaat et al., 2014; Tang et al., 2021; Tong et al., 2011). In this qualitative study of all organ transplant types, it was found that barriers to immunosuppressant adherence were largely consistent across the different transplant cohorts, and importantly, facilitators of good adherence were common to all transplant types, suggesting that interventions to target non-adherence may be transferable across transplant patient cohorts. Additionally, this study reported practical strategies both used and recommended by participants in addition to guidance for new transplant recipients to facilitate good adherence, which has not been captured by similar studies previously.

### 4.1. Directions for future research

Despite widespread acknowledgement of non-adherence as a multi-dimensional concept, most interventions designed to improve medication adherence in both transplant populations and wider health conditions focus on modifiable factors related to the implementation phase of medication taking, which largely tackle non-intentional non-adherence behaviours, such as forgetting or lack of education, and ultimately show limited effectiveness for improving adherence (Nieuwlaat et al., 2014). Our findings show a more dynamic picture of adherence behaviour persisting over time in transplant patients, including a number of processes related to intentional non-adherence, such as inadequate formal psychological support, a lack of social support, the management of medication side effect burden, and competing domestic responsibilities. Triangulation of findings from this study with the most recent systematic review of the effectiveness of interventions to improve immunosuppressant medication adherence (Nieuwlaat et al., 2014), best practice guidelines for adherence measurement (Vrijens et al., 2012) and identification of relevant health behaviour change techniques (Michie et al., 2013) will provide a comprehensive framework from which to design a patient-centred multi-component intervention tailored to patient need, which can provide real-world effectiveness for improving adherence behaviour.

It is also worth noting that the most common age group of participants in our study (35–44 years old) is younger than that of participants in other transplantation studies, as transplant recipients are usually aged 50 years or older (Beerli et al., 2022; Daly et al., 2020), although in Ireland, older cohorts receiving transplants are becoming more common (Bourkas & Achille, 2023). Future research could examine current age differences among transplant recipients in greater detail.

Lastly, no female heart transplant recipients participated in the study, possibly because heart transplants have historically been more common among men in Ireland (Healy et al., 2006); nonetheless, future research should seek to include female heart transplant recipients to assess whether there are differences and/or similarities in their attitudes and experiences of immunosuppressant medication adherence.

### 4.2. Study strengths and limitations

There has been a call for uniformity in the way that nonadherence behaviour is characterised in order to design effective interventions (Vrijens et al., 2016). This study is the first to apply the ABC taxonomy for medication adherence to frame facilitators and barriers to good adherence in terms of their relation to phases of adherence behaviour in a transplant population. This approach facilitates thoughtful identification of the phases that future interventions need to target in order to improve patients’ willingness to persist in taking medications with substantial side effects and potential harms. The sample size of this study may be considered small; however, data saturation principles in qualitative research outline that a larger sample size does not equate to richer qualitative data (Braun & Clarke, 2021; Fusch & Ness, 2015) and that data saturation can occur when 9–17 participants are interviewed (Hennink & Kaiser, 2022; Vasileiou et al., 2018) and in this analysis, no new themes were coded in later interviews; therefore, an assumption of data saturation was taken. Further, the participant yield through recruitment in the outpatient setting was slow. The authors hypothesise that the research topic may have been prohibitive to participant recruitment: there is an inherent social pressure for transplant recipients to have enormous gratitude towards their donor (Bourkas & Achille, 2023), and previous research has highlighted that non-adherent patients are unlikely to participate in a research study on medication non-adherence for fear of judgement for engaging in a behaviour that could harm the success of their transplant (Shemesh & LaPointe Rudow, 2021).

## 5. Conclusion

Patient-reported facilitators and barriers to medication adherence reveal the complex interplay of individual, social and systemic factors that influence medicine-taking behaviour in transplant recipients. Incorporating a medication schedule into daily life incurs psychological and practical consequences that can further impact social and personal well-being if inadequately managed. Regular assessment of mental health status post-transplant and encouragement to seek peer social support is required. Physician-directed access to psychological supports, such as counselling and peer support services for patients, is also pivotal to reduce both feelings of isolation and non-adherence risks. Facilitating access to appropriate psychological support is also necessary for patients to establish a sense of belonging among fellow transplant recipients, help them foster self-acceptance and encourage lifelong adherence to immunosuppressive therapy (persistence). Future patient-centred interventions to improve medication adherence must reflect this complex matrix of barriers, particularly the incorporation of adequate psychological and social support.

## Supplementary Material

Supplementary material Patient Semi Structured Interview Questions.docx

## Data Availability

Data supporting the findings of this study are available from the corresponding author, CD (following approval from LM), upon reasonable request.

## Author declaration statement

This manuscript has been read and approved by all named authors; CD, AW, FD, PC, CO'S, AM, AD, PR and LM. There are no other persons who satisfied the authorship criteria but are not listed. We thereby confirm that the order of the authors listed in this manuscript have been approved by all of us.

## Studies with no animal subjects

This article does not contain any studies with animals performed by any of the authors.

## References

[cit0001] Araya, A. A., & Tasnif, Y. (2023). *Tacrolimus*. StatPearls Publishing.

[cit0002] Beerli, N, Denhaerynck, K, Binet, I, Dahdal, S, Dickenmann, M, Golshayan, D, Hadaya, K., Huynh-Do, U., Schnyder, A., De Geest, S. M., & Mauthner, O. (2022). Age at time of kidney transplantation as a predictor for mortality, graft loss and self-rated health status: Results from the Swiss transplant cohort study. *Transplant International*, *35*, 10076. 10.3389/ti.2021.10076 35185365 PMC8842256

[cit0003] Bendersky, V. A., Saha, A., Sidoti, C. N., Ferzola, A., Downey, M., Ruck, J. M., Vanterpool, K. B., Young, L., Shegelman, A., Segev, D. L., & Levan, M. L. (2023). Factors impacting the medication “Adherence Landscape” for transplant patients. *Clinical Transplantation*, *37*(6),e14962. 10.1111/ctr.14962 36950850 PMC10272095

[cit0004] Bourkas, S., & Achille, M. (2023). The psychosocial adjustment of kidney recipients across donation contexts. *Journal of Health Psychology*, *28*(11), 1011–1023. 10.1177/13591053221149780 36688379 PMC10492421

[cit0005] Braun, V., & Clarke, V. (2006). Using thematic analysis in psychology. *Qualitative Research in Psychology*, *3*(2), 77–101. 10.1191/1478088706qp063oa

[cit0006] Braun, V., & Clarke, V. (2021a). To saturate or not to saturate? Questioning data saturation as a useful concept for thematic analysis and sample-size rationales. *Qualitative Research in Sport, Exercise and Health*, *13*(2), 201–216. 10.1080/2159676X.2019.1704846

[cit0007] Braun, V., & Clarke, V. (2021b). One size fits all? What counts as quality practice in (reflexive) thematic analysis? *Qualitative Research in Psychology*, *18*(3), 328–352. 10.1080/14780887.2020.1769238

[cit0008] Butler, J. A, Roderick, P., Mullee, M., Mason, J. C., & Peveler, R. C. (2004). Frequency and impact of nonadherence to immunosuppressants after renal transplantation: A systematic review. *Transplantation*, 77(5), 769–776. 15021846 10.1097/01.tp.0000110408.83054.88

[cit0009] Colmenero, J., Gastaca, M., Martínez-Alarcón, L., Soria, C., Lázaro, E., & Plasencia, I. (2024). Risk factors for non-adherence to medication for liver transplant patients: An umbrella review. *Journal of Clinical Medicine*, *13*(8), 2348. 10.3390/jcm13082348 38673620 PMC11051511

[cit0010] Corr, M., Walker, A., Maxwell, A. P., & McKay, G. J. (2025). Non-adherence to immunosuppressive medications in kidney transplant recipients–A systematic scoping review. *Transplantation Reviews*, *39*(1), 100900. 10.1016/j.trre.2024.100900 39642406

[cit0011] Daly, A., Cremen, S., & Healy, D. G. (2020). The impact of the new state body for transplantation on lung transplantation waiting list mortality. *Irish Medical Journal*, *113*(2), 19. 32383577

[cit0012] De Geest, S., Zullig, L. L., Dunbar-Jacob, J., Helmy, R., Hughes, D. A., Wilson, I. B., & Vrijens, B. (2018). ESPACOMP medication adherence reporting guideline (EMERGE). *Annals of Internal Medicine*, *169*(1), 30–35. 10.7326/M18-0543 29946690 PMC7643841

[cit0013] Denhaerynck, K., Berben, L., Dobbels, F., Russell, C. L., Crespo-Leiro, M. G., Poncelet, A. J., & De Geest, S. (2018). Multilevel factors are associated with immunosuppressant nonadherence in heart transplant recipients: The international BRIGHT study. *American Journal of Transplantation*, *18*(6), 1447–1460. 10.1111/ajt.14611 29205855 PMC6001479

[cit0014] Denhaerynck, K., Dobbels, F., Cleemput, I., Desmyttere, A., Schaub, S., Schafer-Keller, P., & De Geest, S. (2005). Prevalence, consequences, and determinants of nonadherence in adult renal transplant patients: A literature review. *Transplant International*, *18*(10), 1121–1133. 10.1111/j.1432-2277.2005.00176.x 16162098

[cit0015] Denhaerynck, K., Dobbels, F., Košťálová, B., & De Geest, S. (2023). Psychometric properties of the BAASIS: A meta-analysis of individual participant data. *Transplantation*, *107*(8), 1795–1809. 10.1097/TP.0000000000004574 36949037 PMC10358438

[cit0016] Dew, M. A., & DeVito Dabbs, A. J. (2016). Harnessing the power of qualitative research in transplantation. *American Journal of Kidney Diseases*, *67*(3), 357–359. 10.1053/j.ajkd.2015.12.010 26916372

[cit0017] Dew, M. A., DiMartini, A. F., De Vito Dabbs, A., Myaskovsky, L., Steel, J., Unruh, M., Switzer, G. E., Zomak, R., Kormos, R. L., & Greenhouse, J. B. (2007). Rates and risk factors for nonadherence to the medical regimen after adult solid organ transplantation. *Transplantation*, *83*(7), 858–873. 10.1097/01.tp.0000258599.65257.a6 17460556

[cit0018] DiMatteo, M. R., Haskard-Zolnierek, K. B., & Martin, L. R. (2012). Improving patient adherence: A three-factor model to guide practice. *Health Psychology Review*, *6*(1), 74–91. 10.1080/17437199.2010.537592

[cit0019] Fusch, P., & Ness, L. (2015). Are we there yet? Data saturation in qualitative research. *The Qualitative Report*, *20*(9), 1408–1416. 10.46743/2160-3715/2015.2281

[cit0020] Griva, K., Neo, H. L. M., & Vathsala, A. (2018). Unintentional and intentional non-adherence to immunosuppressive medications in renal transplant recipients. *International Journal of Clinical Pharmacy*, *40*(5), 1234–1241. 10.1007/s11096-018-0652-6 29872960

[cit0021] Healy, D. G., Akbar, M. T., Baktiari, N., Egan, J. J., Mahon, N., Veerasingam, D., McCarthy, J., Hurley, J., Neligan, M., & Wood, A. E. (2006). The first 20 years of heart transplantation in Ireland. *Irish Journal of Medical Science*, *175*(1), 5–9. 10.1007/BF03168991 16615220

[cit0022] Hennink, M., & Kaiser, B. N. (2022). Sample sizes for saturation in qualitative research: A systematic review of empirical tests. *Social Science & Medicine*, *292*, 114523. 34785096 10.1016/j.socscimed.2021.114523

[cit0023] Hu, L., Lingler, J. H., Sereika, S. M., Burke, L. E., Malchano, D. K., DeVito Dabbs, A., & Dew, M. A. (2017). Nonadherence to the medical regimen after lung transplantation: A systematic review. *Heart & Lung: The Journal of Critical Care*, *46*(3), 178–186. 10.1016/j.hrtlng.2017.01.006 28187909

[cit0024] Hussain, T., Nassetta, K., & Badawy, S. M. (2021). Adherence to immunosuppression medications among heart transplant recipients: Challenges, opportunities, and potential role of digital approaches in the COVID-19 era. *Journal of Cardiovascular Development and Disease*, *8*(6), 68. 10.3390/jcdd8060068 34200823 PMC8230436

[cit0025] Jamieson, N. J., Hanson, C. S., Josephson, M. A., Gordon, E. J., Craig, J. C., Halleck, F., Budde, K., & Tong, A. (2016). Motivations, challenges, and attitudes to self-management in kidney transplant recipients: A systematic review of qualitative studies. *American Journal of Kidney Diseases*, *67*(3), 461–478. 10.1053/j.ajkd.2015.07.030 26372087

[cit0026] Kim, H., Sefcik, J. S., & Bradway, C. (2017). Characteristics of qualitative descriptive studies: A systematic review. *Research in Nursing & Health*, *40*(1), 23–42. 10.1002/nur.21768 27686751 PMC5225027

[cit0027] Kupiec-Weglinski, J. W. (2022). Grand challenges in organ transplantation. *Front Transplant*, *1*, 10.3389/frtra.2022.897679 PMC11235338 38994397

[cit0028] Low, J. K., Williams, A., Manias, E., & Crawford, K. (2015). Interventions to improve medication adherence in adult kidney transplant recipients: A systematic review. *Nephrology Dialysis Transplantation*, *30*(5), 752–761. 10.1093/ndt/gfu204 24950938

[cit0029] Mellon, L., Doyle, F., Hickey, A., Ward, K. D., de Freitas, D. G., McCormick, P. A., Mellon, L., de Freitas, D.G., O'Connell, O., & Conlon, P. (2022). Interventions for increasing immunosuppressant medication adherence in solid organ transplant recipients. *Cochrane Database of Systematic Reviews*, *2022*, 10.1002/14651858.CD012854.pub2 PMC9466987 36094829

[cit0030] Mellon, L., Doyle, F., Hickey, A., Ward, K. D., de Freitas, D. G., McCormick, P. A., O'Connell, O., & Conlon, P. (2017). Interventions for improving medication adherence in solid organ transplant recipients. *Cochrane Database of Systematic Reviews*, 2017(12, CD012854. 10.1002/14651858.CD012854 PMC9466987 36094829

[cit0031] Michie, S., Richardson, M., Johnston, M., Abraham, C., Francis, J., Hardeman, W., Eccles, M. P., Cane, J., & Wood, C. E. (2013). The behavior change technique taxonomy (v1) of 93 hierarchically clustered techniques: Building an international consensus for the reporting of behavior change interventions. *Annals of Behavioral Medicine*, *46*(1), 81–95. 10.1007/s12160-013-9486-6 23512568

[cit0032] Moran, C., Doyle, F., Sulaiman, I., Bennett, K., Greene, G., Molloy, G. J., Reilly, R. B., Costello, R. W., & Mellon, L. (2017). The INCATM (Inhaler Compliance AssessmentTM): A comparison with established measures of adherence. *Psychology & Health*, *32*(10), 1266–1287. 10.1080/08870446.2017.1290243 28276739

[cit0033] Mukhtar, O., Weinman, J., & Jackson, S. H. D. (2014). Intentional non-adherence to medications by older adults. *Drugs & Aging*, *31*(3), 149–157. 10.1007/s40266-014-0153-9 24566876

[cit0034] Naeem, M., Ozuem, W., Howell, K., & Ranfagni, S. (2023). A step-by-step process of thematic analysis to develop a conceptual model in qualitative research. *International Journal of Qualitative Methods*, *22*, 16094069231205789. 10.1177/16094069231205789

[cit0035] Nevins, T. E., Nickerson, P. W., & Dew, M. A. (2017). Understanding medication nonadherence after kidney transplant. *Journal of the American Society of Nephrology*, *28*(8), 2290–2301. 10.1681/ASN.2017020216 28630231 PMC5533244

[cit0036] Nieuwlaat, R., Wilczynski, N., Navarro, T., Hobson, N., Jeffery, R., Keepanasseril, A., Agoritsas, T., Mistry, N., Iorio, A., Jack, S., Sivaramalingam, B., Iserman, E., Mustafa, R. A., Jedraszewski, D., Cotoi, C., & Haynes, R. B. (2014). Interventions for enhancing medication adherence. *Cochrane Database of Systematic Reviews*, *2014*(11), CD000011. 10.1002/14651858.CD000011.pub4 25412402 PMC7263418

[cit0037] Organ Donation Transplant Ireland. (2020). *Organ Donation Transplant Ireland: Annual Report 2020*. Health Service Executive.

[cit0038] Organ Donation Transplant Ireland. (2021). *Organ Donation Transplant Ireland: Annual Report 2021*. Health Service Executive.

[cit0039] Organ Donation Transplant Ireland. (2022). *Organ Donation Transplant Ireland: Annual Report 2022*. Health Service Executive.

[cit0040] Organ Donation Transplant Ireland. (2023). *Organ Donation Transplant Ireland: Annual Report 2023*. Health Service Executive.

[cit0041] O'Regan, J. A., Canney, M., Connaughton, D. M., O'Kelly, P., Williams, Y., Collier, G., deFreitas, D. G., O'Seaghdha, C. M., & Conlon, P. J. (2016). Tacrolimus trough-level variability predicts long-term allograft survival following kidney transplantation. *Journal of Nephrology*, *29*(2), 269–276. 10.1007/s40620-015-0230-0 26374111

[cit0042] Pinsky, B. W., Takemoto, S. K., Lentine, K. L., Burroughs, T. E., Schnitzler, M. A., & Salvalaggio, P. R. (2009). Transplant outcomes and economic costs associated with patient noncompliance to immunosuppression. *American Journal of Transplantation*, *9*(11), 2597–2606. 10.1111/j.1600-6143.2009.02798.x 19843035

[cit0043] Shemesh, E., & LaPointe Rudow, D. (2021). Perspectives of solid organ transplant recipients on taking medications: Valuable research, just the beginning. *American Journal of Transplantation*, *21*(10), 3221–3222. 10.1111/ajt.16620 33891800

[cit0044] Springfield-Trice, S., Reddy, G., Joyce, C., Garcia, B. M., Shah, P., Agbor-Enoh, S., & Valantine, H. (2025). Barriers to immunosuppressant medication adherence in thoracic transplant recipients: Initial findings. *International Journal of Environmental Research and Public Health*, *22*(7), 1090. 10.3390/ijerph22071090 40724157 PMC12294800

[cit0045] Stuber M. L. Psychiatric aspects of organ transplantation in children and adolescents. *Psychosomatics*. 1993;*34*(5):379–387. 10.1016/S0033-3182(93)71840-X 8140186

[cit0046] Tang, J., Kerklaan, J., Wong, G., Howell, M., Scholes-Robertson, N., Guha, C., Kelly, A., & Tong, A. (2021). Perspectives of solid organ transplant recipients on Medicine-taking: Systematic review of qualitative studies. *American Journal of Transplantation*, *21*(10), 3369–3387. 10.1111/ajt.16613 33866675

[cit0047] Tong, A., Sainsbury, P., & Craig, J. (2007). Consolidated criteria for reporting qualitative research (COREQ): A 32-item checklist for interviews and focus groups. *International Journal for Quality in Health Care*, *19*(6), 349–357. 10.1093/intqhc/mzm042 17872937

[cit0048] Tong, A., Howell, M., Wong, G., Webster, A. C, Howard, K., & Craig, J. C. (2011). The perspectives of kidney transplant recipients on Medicine taking: A systematic review of qualitative studies. *Nephrology Dialysis Transplantation*, *26*(1), 344–354. 10.1093/ndt/gfq376 20584734

[cit0049] Vasileiou, K., Barnett, J., Thorpe, S., & Young, T. (2018). Characterising and justifying sample size sufficiency in interview-based studies: Systematic analysis of qualitative health research over a 15-year period. *BMC Medical Research Methodology*, *18*(1), 148. 10.1186/s12874-018-0594-7 30463515 PMC6249736

[cit0050] Vrijens, B., De Geest, S., Hughes, D. A., Przemyslaw, K., Demonceau, J., Ruppar, T., Dobbels, F., Fargher, E., Morrison, V., Lewek, P., Matyjaszczyk, M., Mshelia, C., Clyne, W., Aronson, J. K., & Urquhart, J. (2012). A new taxonomy for describing and defining adherence to medications. *British Journal of Clinical Pharmacology*, *73*(5), 691–705. 10.1111/j.1365-2125.2012.04167.x 22486599 PMC3403197

[cit0051] Vrijens, B., Dima, A. L., Van Ganse, E., van Boven, J. F. M., Eakin, M. N., Foster, J. M., de Bruin, M., Chisholm, A., & Price, D. (2016). What we mean when we talk about adherence in respiratory medicine. *Journal of Allergy and Clinical Immunology*, *4*(5), 802–812. 10.1016/j.jaip.2016.05.019 27587314

