# Supplementary material for: ‘‘That's just part of having a transplant, that's the price you pay” Patient-reported facilitators and barriers to immunosuppressant medication adherence among transplant recipients: a qualitative study
Source: Health Psychol Behav Med. 2026 Mar 12;14(1):2641868. doi: 10.1080/21642850.2026.2641868 (PMC12983805; doi:10.1080/21642850.2026.2641868)
Supplement: Supplementary material — Patient Semi Structured Interview Questions.docx [file RHPB_A_2641868_SM5729.docx]

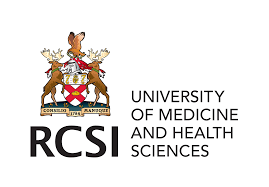
**The interviewer will greet the participant and introduce themselves. The participant will be thanked for their willingness to contribute to the study and will be given an overview of the study and reminded about the purpose of the interview. They will also be informed that they can pause and/or terminate the interview at any stage without question and that they have the right to review their transcript after it is produced by the research assistant. Similarly, if they wish to withdraw from the study they can do so before the write-up and publication stages. Before commencing the interview, the participant will be asked if they have any questions for the interviewer.**

Q1. How long have you been taking your prescribed immunosuppressant medications and what has your experience been like you taking them every day?

Q2. Has any health care professional involved in your care supported you in taking your immunosuppressant medications?

Q3. Have you endured any side effects from taking your immunosuppressant medications? If yes, what has that been like for you? (frequency, type, duration, impact).

Q4. Do you have any strategies that help you take your immunosuppressant medications?

Q5. What difficulties have you experienced in your day-to-day life from taking your immunosuppressants?

Q6. Have you ever accidentally forgot or intentionally decided not to take your immunosuppressant medications?

Q7. Are there any strategies that you feel would help you and other patients to take their immunosuppressant medications as prescribed?

Q8. What format do you think would work best for patients to ensure that they use strategies that would help them take their medication as prescribed? (Being told in-person, mobile app, website, leaflet etc.).

Q9. Do you have any suggestions for improving communication between health care professionals and patients about taking their medications as prescribed?

Q10. Is there anything you would like to add before we finish up?
